# Enhanced docetaxel therapeutic effect using dual targeted SRL-2 and TA1 aptamer conjugated micelles in inhibition Balb/c mice breast cancer model

**DOI:** 10.1038/s41598-024-75042-8

**Published:** 2024-10-19

**Authors:** Yasamin Davatgaran Taghipour, Amir Zarebkohan, Roya Salehi, Mehdi Talebi, Reza Rahbarghazi, Monireh Khordadmehr, Sharareh Khavandkari, Fahimeh Badparvar, Vladimir P. Torchilin

**Affiliations:** 1 Department of Medical Nanotechnology, School of Advanced Medical Sciences, Tabriz University of Medical Sciences, Tabriz, 516661-4733 Iran; 2 Drug Applied Research Center, Department of Medical Nanotechnology, School of Advanced Medical Sciences, Tabriz University of Medical Sciences, Tabriz, 516661-4733 Iran; 3 Clinical Research Development Unite of Tabriz Valiasr Hospital, Tabriz University of Medical Sciences, Tabriz, 51666-18559 Iran; 4 Department of Applied Cell Sciences, Faculty of Advanced Medical Sciences, Tabriz University of Medical Sciences, Tabriz, Iran; 5 Stem Cell Research Center, Department of Applied Cell Sciences, Faculty of Advanced Medical Sciences, Tabriz University of Medical Sciences, Tabriz, Iran; 6 Department of Pathobiology, Faculty of Veterinary Medicine, University of Tabriz, Tabriz, Iran; 7 Department of Animal Biology, Faculty of Natural Sciences, University of Tabriz, Tabriz, Iran; 8 Department of Organic Chemistry, Faculty of Chemistry, Urmia University, Urmia, Iran; 9 Center for Pharmaceutical Biotechnology and Nanomedicine, Department of Chemical Engineering, Northeastern University, Boston, USA

**Keywords:** Drug resistance, DTX, Delivery, Smart, GSH/pH responsive, TA1 aptamer, SRL-2, Biochemistry, Biological techniques, Cancer, Chemistry, Materials science

## Abstract

Effective targeting and delivery of large amounts of medications into the cancer cells enhance their therapeutic efficacy through saturation of cellular defensive mechanisms, which is the most privilege of nano drug delivery systems (NDDS) compared to traditional approaches. Herein, we designed dual-pH/redox responsive DTX-loaded poly (*β*-amino ester) (PBAS) micelles decorated with a chimeric peptide and TA1 aptamer. In vitro and in vivo results demonstrated that the designed nanoplatform possessed an undetectable nature in the blood circulation, but after exposure to the tumor microenvironment (TME) of 4T1 breast cancer, it suddenly changed into dual targeting nanoparticles (NPs) (containing two ligands, SRL-2 and TA1 aptamer). The dual targeting NPs destruction in the high GSH and low pH conditions of the cancer cells led to amplified DTX release (around 70% at 24 h). The IC50 value of DTX-loaded MMP-9 sensitive heptapeptide/TA1 aptamer-modified poly (*β*-amino ester) (MST@PBAS) micelles and free DTX after 48 h of exposure was determined to be 1.5 µg/ml and 7.5 µg/ml, respectively. The nano-formulated DTX exhibited cytotoxicity that was 5-fold stronger than free DTX (Pvalue˂0.001). Cell cycle assay test results showed that following exposure to MST@PBAS micelles, a considerable rise in the sub G1 population (48%) suggested that apoptosis by cell cycle arrest had occurred. DTX-loaded MST@PBAS micelles revealed significantly higher (Pvalue ˂ 0.001) levels of early apoptosis (59.8%) than free DTX (44.7%). Interestingly, in vitro uptake studies showed a significantly higher TME accumulation of dual targeted NPs (6-fold) compared to single targeted NPs (Pvalue < 0.001) which further confirmed by in vivo biodistribution and fluorescent TUNEL assay experiments. NPs treated groups demonstrated notable tumor growth inhibition in 4T1 tumor bearing Balb/c mice by only 1/10th of the DTX therapeutic dose (TD) as a drug model. In conclusion, cleverly designed nanostructures here demonstrated improved anticancer effects by enhancing tumor targeting, delivering chemotherapeutic agents more accurately, promoting drug release, reducing the therapeutic dosage, and lowering side effects of anticancer drugs.

## Introduction

Chemotherapeutic drugs are a cornerstone in the treatment of cancer, produced to kill or inhibit the growth of cancerous cells. However, they often come with significant adverse effects on the human body due to their lack of specificity, impacting both cancerous and healthy cells^1,2^. Their adverse effects include hematologic toxicity (bone marrow suppression)^3^, gastrointestinal toxicity (nausea and vomiting)^4^, alopecia (hair loss)^5^, cardiotoxicity^6^, neurotoxicity^7^, renal, and hepatic toxicity^8^ arise from off-targeting and hydrophobic nature. Breast cancer, one of the most common cancers affecting women worldwide, is often treated with chemotherapeutic drugs. Furthermore, drug resistance remains a significant challenge, limiting the effectiveness of chemotherapy^9^.

Genetic mutations and epigenetic changes can cause drug resistance. For example, gene amplification like HER2 increases resistance to trastuzumab, while mutations in drug targets, such as estrogen receptor mutations, can reduce the effectiveness of hormone therapies like tamoxifen. Additionally, overexpression of drug efflux pumps like P-glycoprotein can lower the concentration of chemotherapeutic drugs inside cancer cells, and changes in membrane phospholipids can hinder drug penetration^10,11^. Another drug resistance mechanism is altered drug metabolism which is changes in the expression or activity of enzymes involved in drug metabolism can lead to increased detoxification and elimination of drugs. For instance, increased activity of glutathione S-transferase can neutralize alkylating agents used in chemotherapy^12^. DNA repair mechanisms which mean enhanced DNA repair capabilities can counteract the DNA-damaging effects of chemotherapy drugs like cisplatin. For example, upregulation of the BRCA1 and BRCA2 genes involved in homologous recombination repair can confer resistance^13^. Also, apoptosis evasion through overexpression of anti-apoptotic proteins (e.g., Bcl-2) or downregulation of pro-apoptotic proteins (e.g., Bax), reducing the effectiveness of drugs that induce cell death^14^. Given the complex mechanisms of resistance, alternative treatment strategies are essential to improve outcomes for breast cancer patients.

Smart drug delivery systems aim to improve the specificity and efficacy of chemotherapeutic drugs while minimizing their adverse effects. By targeting drug delivery more precisely to cancer cells, these innovations hold the potential to significantly reduce the collateral damage to healthy tissues and improve overall treatment outcomes for patients^15^. Delivery of drug concentrations more than cellular drug resistance capacitance is another benefit of nano drug delivery system which indirectly neutralizes defensive mechanisms. In smart transformable nanoparticles (NPs), different inherent pathological properties of tumor microenvironment (TME), such as pH, redox system, reactive oxygen free radical species, and extracellular matrix metalloproteinases (MMPs), have been employed^16^. Recently, some intelligent NPs have been developed that possess chimeric MMP-sensitive sequences in their structures, which were activated after exposure to the MMPs in TME^17^ and result in enhanced tumor penetration ability of the NPs. To prevent the NPs involvement in protein corona formation, we concealed the TA1 aptamers between a cleavable stealth chimera peptide (CSRLSLPGSSSKpalmSSS) which consistead of two peptides^18^. Protein corona (PC) is the binding of proteins in biological fluids to the surface of NPs, that subsequently alters the biological nature, efficacy and toxicity profiles of NPs. Additionally, the functionality of well-known ligands used in pharmaceuticals, especially aptamers, can be completely changed depending on their nature. For example, TA1 aptamer, one of the best ligands discovered in recent years, was abandoned in clinical Phase 2 due to the loss of efficacy following protein binding in the blood^19–21^.

In this study, by designing an intelligent system, we were able to maintain the efficacy of this aptamer as a ligand at the tumor site (by shielding the ligand from contact with blood proteins through encapsulation between fusion proteins) and, at the tumor site, facilitate targeting and cellular uptake dependent on this aptamer and peptide (SRL-2) through interaction with cancer cells.

**Scheme 1:**
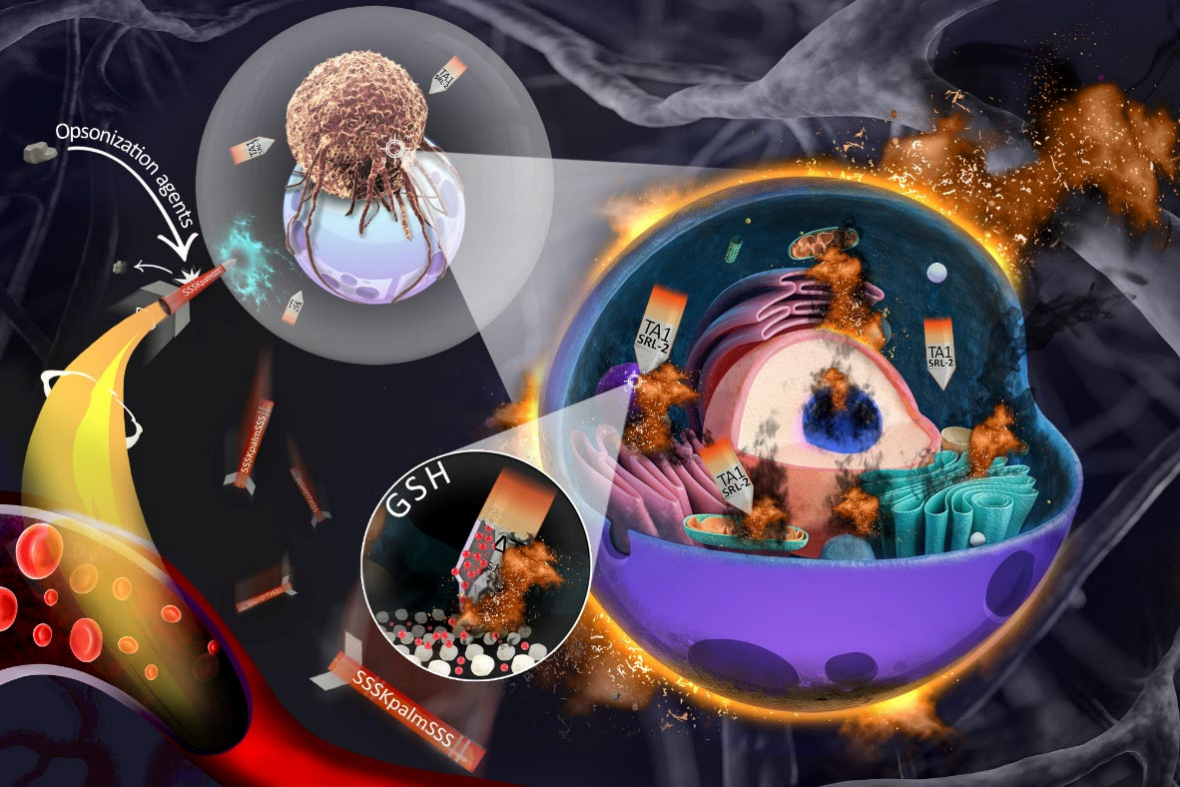
Schematic illustration of the work which showed biologically transformable NPs (aptamer and peptide-modified DTX-loaded pH and glutathione (GSH) dual responsive polymeric micelles) with altered performance after exposure to overexpressed MMP-9 in breast cancer TME. The synthesized NPs generally exhibit relatively neutral biological activity in the bloodstream and show minimal interaction with blood proteins (especially opsonins), as noted in the earlier article. However, upon entering the tumor microenvironment, and following the breakdown of the fusogenic peptide, the hidden aptamers (TA1) between these peptides, along with the remaining peptide sequence (SRL-2), suddenly become exposed. These aptamers alongside of SRL-2 peptide (10 amino acid remaining after cleavage of fusogenic peptide) then synergistically recognize the CD44 and LRP-1, respectively on cancer cells, demonstrating a significantly high cellular uptake efficiency. Essentially, our initial nanoparticles function like a shuttle, as depicted, that upon reaching the tumor boundary (illustrated by the gray bubble around the tumor in the schematic), loses part of its structure. The remaining part of the structure, resembling a missile cap with dual targeting ends, identifies the target cell receptors and effectively delivers the chemotherapeutic drug with high efficacy (at 1/10th of the therapeutic concentration) to the cancer cells.

The results confirmed that after exposure to TME of breast cancer, chimera peptide cleaved and remained amino acid sequence and TA1 aptamers simultaneously and synergistically target the cancer cells. Herein, we used this smart structure for delivery of DTX to the breast cancer and showed that 1/10th of the therapeutic dosage (TD) of DTX effectively ceased tumor growth in 4T1 tumor-bearing Balb/c mice (Scheme 1).

## Materials and methods

### Materials

Maleimide-polyethylene glycol-N-hydroxysuccinimide (MAL-PEG-NHS) was purchased from JenKem Technology (U.S.A), DTX was purchased from Tabriz university of medical sciences pharmacy (Iran). 1,4-Butanediol Diacrylate, Bis(2-methacryloyl) oxyethyl disulfide, Ethylenediamine, 3-Amino-1- propanol, rhodamine B dye, were purchased from Sigma-Aldrich (Germany). 5’- CCAAGGCCTGCAAGGGAACCAAGGACACAGTTTTTTTTTT-SH-3’ sequence which belongs to CD44 aptamer (TA1) was bought from Eurofins genomics company (Germany). C-SRLSLPGSSSK-palm-SSSS peptide sequence was bought from GL Biochem (China).

#### Cells and culture environment

The Pasteur Institution (Iran) provided the 4T1 cell line, which was then cultured in Gibco’s (U.S.A.) Roswell Park Memorial Institute (RPMI) 1640 medium with 10% FBS and 1% penicillin-streptomycin supplements at 37 °C in a CO2 (5%) moist environment.

#### Animal model

The National Institutes of Health’s (NIH) recommendations for the handling, feeding, and use of laboratory animals were followed in all in vivo tests. Animal welfare and usage were approved by the Ethics Committee of the Tabriz University of Medical Sciences, Iran with ethical approval code of IR.TBZMED.VCR.REC.1398.158, and this guideline is based on EU Directive 2010/63/EU of the European parliament and of the council of 22 September 22, 2010 on the protection of animals used for scientific purposes. We confirmed that all methods were performed in accordance with the relevant guidelines and regulations. Female Balb/C mice, aged 4 weeks and weighing 20–25 g, were obtained from the Iranian company Pishro Mehravaran Azma Pars. The mice were housed under standard conditions, and they were allowed to acclimate to their environment before initiating the tumor-bearing animal model. To establish the model, 5 × 10^6^ 4T1 cells were injected subcutaneously beneath the right flank of each mouse. Typically, the tumor reached a size of 50 mm^3^ after approximately ten days, at which point the dual-targeted NPs were administered. The NPs were injected four times with the volume of 200 µL at a dose of 1 mg/kg.

### Synthesis of poly (β-amino ester) polymers

Briefly, Dichloromethane (5 ml) was dissolved in 1,4-butanediol diacrylate (2.4 mmol, 2262 l) in the three-necked round-bottom flask while being kept in an ice bath and refluxing. Then, 150 µl of 0.6 mmol of bis(2-methacryloyl) oxyethyl disulfide, a crosslinking agent with a disulfide bond, was added to the solution. Then, dropwise addition of 3-amino-1-propanol (2 mmol, 764 µl) was made using a septum-sealed neck. The mixture was continuously bubbled with nitrogen for 15 min. The reaction was then carried out under reflux conditions at a temperature of 90 °C for 24 h. The resulting product, which exhibited a dark yellow color and had a viscous consistency, confirmed the formation of disulfide bonds. The polymeric solution was placed in an ice bath while ethylenediamine (6 ml) was dissolved in DMSO (6 ml) and added dropwise. The final drop was added gradually during the process. The residual reaction was allowed to continue for 24 h at room temperature. The finished product underwent a 48-hour freeze-drying process after that. At each stage of the synthesis, unreacted materials were removed from the environment using a dialysis bag with MWCO of 2000 Da. Following synthesis, the TNBSA test was used in accordance with the manufacturer’s instructions to calculate the number of free amine groups present in the NPs^22^. The calculated amount of MAL-PEG-NHS was added to the polymeric structure once the number of free amine groups had been determined, and it was agitated for 24 h before freeze-drying. Using Ellman’s reagent, the amount of free sulfhydryl groups on the peptides was calculated^23^. The bifunctional PEG-modified PBAS polymer was then conjugated with particular components via the thiol-ene process to create three distinct formulations. Noteworthy to mention that, according to previously reported work, the ratio of modified monomers utilized for obtaining camouflaged micelles (MST@PBAS) was 9:1 (chimer-modified peptide: TA1-modified peptide)^24^. Moreover, the CMC of the formed micelles was achieved at 25 µg mLˉ^1^.

#### Characterization the features of PBAS polymers and MST@PBAS NPs

Proton nuclear magnetic resonance (^1^HNMR) spectra were collected to verify each step of the synthesis. The samples were dissolved in deuterated chloroform (CDCl3) to get the spectra. Dynamic light scattering was used to determine the mean particle size diameter, polydispersity index (PDI), and zeta potential of different micelle formulations (PBAS, MST@PBAS, ). Transmission electron microscopy (TEM) was used to assess the morphology of the final micelle formulation (MST@PBAS).

#### Drug loading study

A solution containing the DTX and polymer in a 5:1 ratio was diluted in 2 ml of DMSO to create DTX-loaded MST@PBAS micelles. Using probe sonication, this solution was then gradually added drop by drop to a 5 ml polyvinyl alcohol (PVA) solution with a concentration of 0.25% (w/v). The resulting mixture underwent purification through dialysis using a membrane with a molecular weight cutoff (MWCO) of 2000 Da. The dialysis process was carried out for 4 h in fresh distilled water. The DTX loads in the center of the polymeric micelles, and the TEM image proves that in the next section. The drug loading and release were evaluated at a wavelength of 231 nm using a UV-vis spectrophotometer. The drug encapsulation efficiency (DEE) was calculated using the following formula:

DEE (%) = $$\:\frac{\text{M}\text{a}\text{s}\text{s}\:\text{o}\text{f}\:\text{d}\text{r}\text{u}\text{g}\:\text{i}\text{n}\:\text{n}\text{a}\text{n}\text{o}\text{c}\text{a}\text{r}\text{r}\text{i}\text{e}\text{r}}{\text{M}\text{a}\text{s}\text{s}\:\text{o}\text{f}\:\text{f}\text{e}\text{e}\text{d}\:\text{d}\text{r}\text{u}\text{g}}$$ ˟ 100

#### Drug release study

Utilizing DTX-loaded MST@PBAS micelles in various media, in vitro drug release tests were performed to evaluate the polymeric micelles’ redox- and pH-responsive behavior. Initially, 2 mg of drug-loaded micelles with a MWCO of 2000 Da were delivered to a dialysis tube. Next, 2 ml of each of four distinct release media: pH 6, pH 6 + 10 mM GSH, pH 7.4 + 10 µM GSH, and pH 7.4 was added to the tubes. To aid in the better release of DTX, a sink solution made up of 97% PBS and 3% methanol was created. The complete release medium was taken out of the dialysis tube at specified intervals (1, 4, 8, 24, 48, 72, 96, and 120 h) in order to measure the amount of medication released at a wavelength of 231 nm. The tube was then filled with the same volume of brand-new media. The following equation was used to compute the proportion of medication released:

Drug release (%) = $$\:\frac{\sum\:_{\text{t}}^{{\uptheta\:}}\left(\text{a}\text{m}\text{o}\text{u}\text{n}\text{t}\:\text{o}\text{f}\:\text{d}\text{r}\text{u}\text{g}\:\text{i}\text{n}\:\text{r}\text{e}\text{l}\text{e}\text{a}\text{s}\text{e}\:\text{m}\text{e}\text{d}\text{i}\text{u}\text{m}\:\text{a}\text{t}\:\text{t}\text{i}\text{m}\text{e}\:\text{t}\right)\:}{\text{a}\text{m}\text{o}\text{u}\text{n}\text{t}\:\text{o}\text{f}\:\text{d}\text{r}\text{u}\text{g}\:\text{l}\text{o}\text{a}\text{d}\text{e}\text{d}\:\text{i}\text{n}\:\text{n}\text{a}\text{n}\text{o}\text{c}\text{a}\text{r}\text{r}\text{i}\text{e}\text{r}}$$

### Intracellular uptake study

4T1 cells were cultured in 6-well plates at a density of 2 × 10^5^ cells/well for quantitative evaluation of the NPs uptake. After a 48-hour incubation period, the cells were treated with various formulations of rhodamine-B loaded NPs. The Rhodamine B-loaded micelles were made by dissolving predetermined amounts of NPs and rhodamine-B in DMSO. This solution was then added dropwise to a PVA 1% w/v solution under probe sonication in the dark. The resulting micelles were then measured for uptake by the cells. Rhodamine-B loaded NPs were analyzed for concentration (0.312, 0.156, and 0.078 µg/mL) and time dependent (30, 90, and 180 min) internalization behavior (3 h). After incubation of NPs with cells for 3 h, the cells were trypsinized and washed with cold PBS three times, subsequently resuspended in a final volume of 500 µl PBS. The cells’ fluorescence output was then measured with a flow cytometer and examined using FlowJo software. Also, for qualitative evaluation of cellular uptake the same number of cells were planted in 6-well plates and cultured for 48 h. Afterwards, Rhodamine-B loaded NPs were introduced and incubated with the cells for 3 h and were examined under a fluorescence microscope (BX51, Olympus, Japan) on glass cover slips.

### Cell viability assay in vitro

In summary, 96-well culture plates were prepared with 10^4^ 4T1 cells in each well and incubated for 48 h. Following this, the cells were exposed to various concentrations of blank micelle free DTX and DTX-loaded MST@PBAS micelles (2.5, 1.25, 0.625, 0.3125, and 0.15625 µg/mL) for an additional 48 h. The viability of blank micelles was investigated in Primary Human Umbilical Vein Endothelial Cells (HUVEC) and Human Fetal Foreskin Fibroblast 2 (HFFF2) cell lines too. After the incubation period, MTT solution (0.5 mg/mL) was added to each well and further incubated for 4 hours. The absorbance of the resulting solution was then measured at 570 nm. Each measurement was performed in triplicate.

### Cell cycle analysis

The 4T1 cells were cultured in 6-well plates at a density of 2 × 10^5^ cells/well, and they were divided into four groups: negative control cells, cells treated with free DTX, cells treated with DTX-loaded MST@PBAS micelles, and cells treated with blank micelles. After 48 h of treatment, the cells were harvested and fixed with 70% cold ethanol. The fixed cells were then incubated at 4ºC for 3–5 days. After the incubation period, the cells were treated with ribonuclease A to digest the RNA and stained in the dark with a propidium iodide solution containing 0.1% Triton-X100. A flow cytometer was used to evaluate the fluorescence evaluation of the stained cells.

### Apoptosis assay

The apoptosis assay was performed using the Ex-bioscience apoptosis detection kit. 4T1 cells were seeded in 6-well plates at a density of 2 × 10^5^ cells per well and incubated for 24 h. Subsequently, the cells were treated with DTX-loaded MST@PBAS micelles and free DTX at their respective IC50 concentrations, which were determined using the MTT assay. The assay procedure followed the instructions provided by the kit manufacturer. The samples were immediately analyzed using a FACScalibur flow cytometer.

### In vivo anti-tumor efficacy

A 4T1 tumor-bearing mice model was used to assess the therapeutic efficacy of DTX-loaded MST@PBAS micelles to free DTX. Six mice from each of three treatment groups were randomly assigned to the mice once the tumor volume reached around 50 mm^3^. The treatments were initiated accordingly. The first group received free DTX as the control, administered intraperitoneally at a dosage of 10 mg/kg. The second group received DTX-loaded MST@PBAS micelles, where the micelles contained 1 mg/kg of DTX. The micelles were also administered intraperitoneally. The third group, serving as the control group, received a physiological saline solution injected at the same volume (200 µl). The NPs dose injection interval was every 4 days for 20 days. Tumor growth was monitored, and the tumor volume was measured every two days for a period of 20 days. The tumor volume was calculated using the formula: V (mm^3^) = 1/2 × L × W1^2^, where L represents the length and W represents the width of the tumor.

### Histological and Immunohistochemical Analysis

We sacrificed mice after treatments through intraperitoneal injection of a high dose of ketamine/xylazine (overdose) according to ethical guidelines. Immediately following euthanasia on the 21st day after injection of the nano-formulations, tissue samples including the tumor mass, lung, liver, kidney, spleen, heart, and brain were obtained for histological investigation. After being fixed in 10% buffered formalin, the samples were paraffin embedded. Three serial slices of roughly 5 μm thickness were taken, and they were each stained with the standard hematoxylin and eosin (H&E) stain^22^. The tissue sections were examined under a light microscope (Olympus-CH30, Japan) to detect any histological abnormalities like vascular congestion, hemorrhage, and necrosis as well as tumor proliferation, invasion, and metastasis. The transferase-mediated deoxyuridine triphosphate-biotin nick end labeling (TUNEL) experiment was carried out using the Elabscience^®^ TUNEL assay kit to assess tumor apoptosis in the various treatment groups. An Olympus BX50 microscope was used to examine the dyed tumor slides.

### Statistical investigation

The results of every experiment were run in triplicate, and they were presented as means standard deviation (SD). Microsoft Excel 2019 or GraphPad Prism software (version 8) were both used for data analysis. One-way analysis of variance (ANOVA) was used for the statistical studies. To indicate statistical significance, the significance threshold was set at *p < 0.05, **p < 0.01, and ***p < 0.001 to denote statistical significance.

## Results and discussion

### Synthesis and characterization of the PBAS polymers

**Fig. 1:**
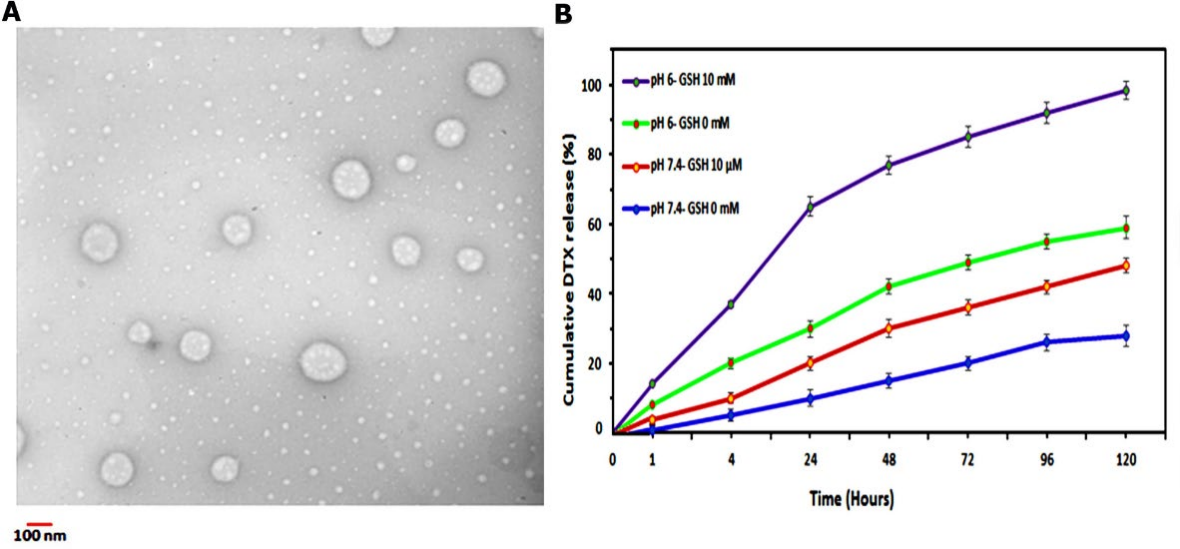
(**A**) The transmission electron microscopy (TEM) image of DTX-loaded MST@PBAS NPs. (**B**) The in vitro drug release profile of DTX from MST@PBAS micelles over 120 h at different conditions: pH 6 with 10 mM GSH (purple), pH 6 (green), pH 7.4 with 10 µM GSH (red), and pH 7.4 (blue).

As previously reported, dual stimuli responsive aptamer and peptide modified PBAS micelles were step by step synthesized and characterized, and TNBSA and Elman’s assay further validated the outcome^24^. As it can be seen in Fig. 1A, dual targeted DTX-loaded micelles demonstrated homogenous, spherical shape with the approximate size of 100 nm which was compatible for the application as in vitro and in vivo drug delivery system.

Also, the critical micelle concentration of the MST@PBAS NPs was obtained 25 g mL^−1^ based on the fluorescence intensity plot^24^. In addition, hydrodynamic diameter and zeta potential of MST@PBAS NPs were 82.26, 100 nm and − 14.5 mV, -6.20 mV before, and after DTX loading respectively, confirming the successful production of NPs. According to principals of successful drug delivery, in which ideal NPs should be relatively negative charged to neutral zeta potential with diameter under 100 nm, DTX-loaded MST@PBAS NPs have been demonstrated suitable physicochemical properties^25^. Moreover, UV-visible analysis was illustrated that encapsulation efficiency of DTX in MST@PBAS micelles was 45 ± 0.9%, which the content of DTX in NPs was measured 1 mg per 5 mg NP.

As intracellular burst release of drugs from NPs has a pivotal role in overcome to drug resistance capability of the cancer cells, the main logical reason behind this concept arises from the fact that every biological motor shows maximum working capacity^26^. Therefore, MDRs are saturated by sudden increase of the concentration of chemotherapeutic agents in the cells, subsequently retained drug molecules have enough time to reach into the nucleus of the cell and destruct DNA structure. In fact, the highly acidic and reductive nature of the cancer cells have been targeted as novel promising cancer treatment strategies in the past decade^27,28^. Synthesized DTX-loaded MST@PBAS NPs behave in pH/redox dependent manner so that around 70% of DTX have been released within 24 h which was remarkable higher than other medias (Fig. 1B). This phenomenon showed the power of synergistic effect of pH and GSH compared to each media alone. For instance, pH 6 and pH 7.4 reached 30% and 10% release in 24 h, respectively which were significantly different from the application of pH and GSH together.

### Cellular internalization study

**Fig. 2:**
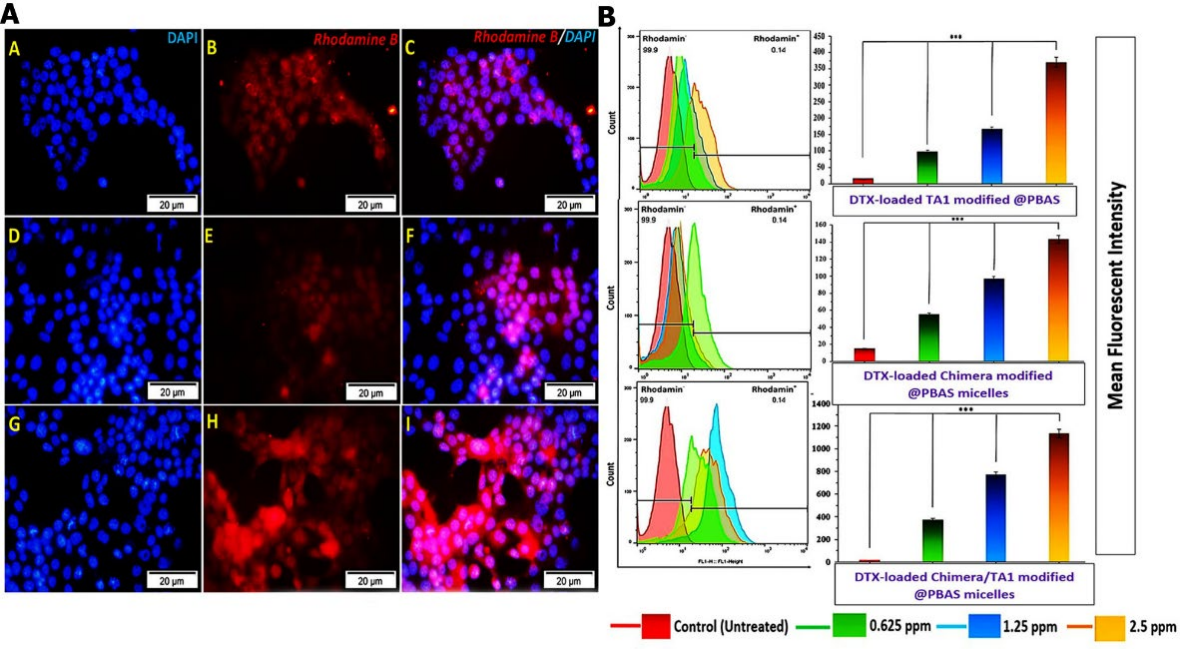
In vitro cellular uptake results. (**A**) Qualitative uptake of different decorations of DTX-loaded nano-micelles; T@PBAS (**A**–**C**), MS@PBAS (**D**–**F**), and MST@PBAS (**G**–**I**) at concentration of 0.625 µg/ml and exposure time of 3 h in 4T1 cells by the fluorescent microscopy. (**B**) Concentration dependent uptake of different decorations of DTX-loaded T@PBAS, MS@PBAS, and MST@PBAS micelles (2.5, 1.25, 0.625 µg/ml) in 4T1 cell line in constant exposure time of 3 h by the flow cytometer.

The cellular uptake efficiency of DTX-loaded MST@PBAS NPs by 4T1 cell lines was evaluated both qualitatively and quantitatively using fluorescent microscopy (Fig. 2A) and flow cytometry (Fig. 2B), respectively. The results obtained from fluorescent microscopy demonstrated that the internalization efficiency of DTX-loaded NPs was significantly higher than control group (Fig. 2A), confirming the excellent 4T1 cells targeting capability, specially in time dependent manner which could be due to the enough time to cleave chimeric peptide by expressed MMP-9 in vitro. We showed that^24^ SRLSLPGSSSK-palm-SSSS peptide act as antifouling coverage on the surface of NPs. However, exposure to breast cancer cell media due to presence of MMP-9 led to breaking the amino acid sequence from SSS sequence in the left side. The remaining 10 amino acids after cleavage, (CSRLSLPGSS, (SRL-2)) act as a strong ligand towards low-density lipoprotein receptor-related protein (LRP-1). LRP-1 extensively expressed on the endothelial cells of blood brain barrier and showed potent transcytosis capability^29–31^. Finally, co-existence of SRL-2 and TA1 synergistically enhanced the efficacy of the targeting and cellular internalization compared to control groups (TA1 or SRL-2 alone)^30,32^. Another evidence which emphasized our conclusion arised from cell uptake of NPs by Raw264.7 cells. More interestingly, results showed that internalization of MST@PBAS NPs decreased by enhancement in the concentration of chimeric peptide in the structure of NPs, especially after PC formation. Due to the absence of the MMP-9 in Raw264.7 cultured media, consequently, antifouling heptapeptide wasn’t cleaved and acted as steric hindrance on the surface of the NPs and inhibited their identification and endocytosis by cell surface receptors^24^. Therefore, DTX loaded MST@PBAS NPs worked as stealth NPs in the body and this feature decreased the required amounts of drug and resulted in diminishing of off-target effects. Also, using two ligands on the surface of NPs with different intracellular delivery mechanisms increased the chance of drug accumulation in the cell and tackled the MDR function in drug resistance. In this regard, qualitative and quantitative flourescent studies showed that DTX-loaded MST@PBAS NPs have been uptaked in time and concentration-dependent manner which were dramatically (5–6 times) higher (MFI 1190) than each aptamer (MFI 350) or peptide (MFI 140) modified formulations (one ligand on the NP) (Fig. 2B). Interestingly, in contrast to our previous work^24^, the uptake ratio of the same NPs changed when we altered the drug molecules (DTX versus salinomycin). This observation further supports the advantage of nano-based drug delivery systems, highlighting their dependence on the physicochemical properties of the nanoparticles. Notably, this adaptable formulation addressed limitations of previous formulations, such as Abraxane™, which primarily enhanced blood circulation time by avoiding initial immune cell detection. Our approach provides a more comprehensive solution by effectively targeting drug delivery^33–35^.

### Cell cycle, apoptosis assay, and Cytotoxicity experiment

**Fig. 3:**
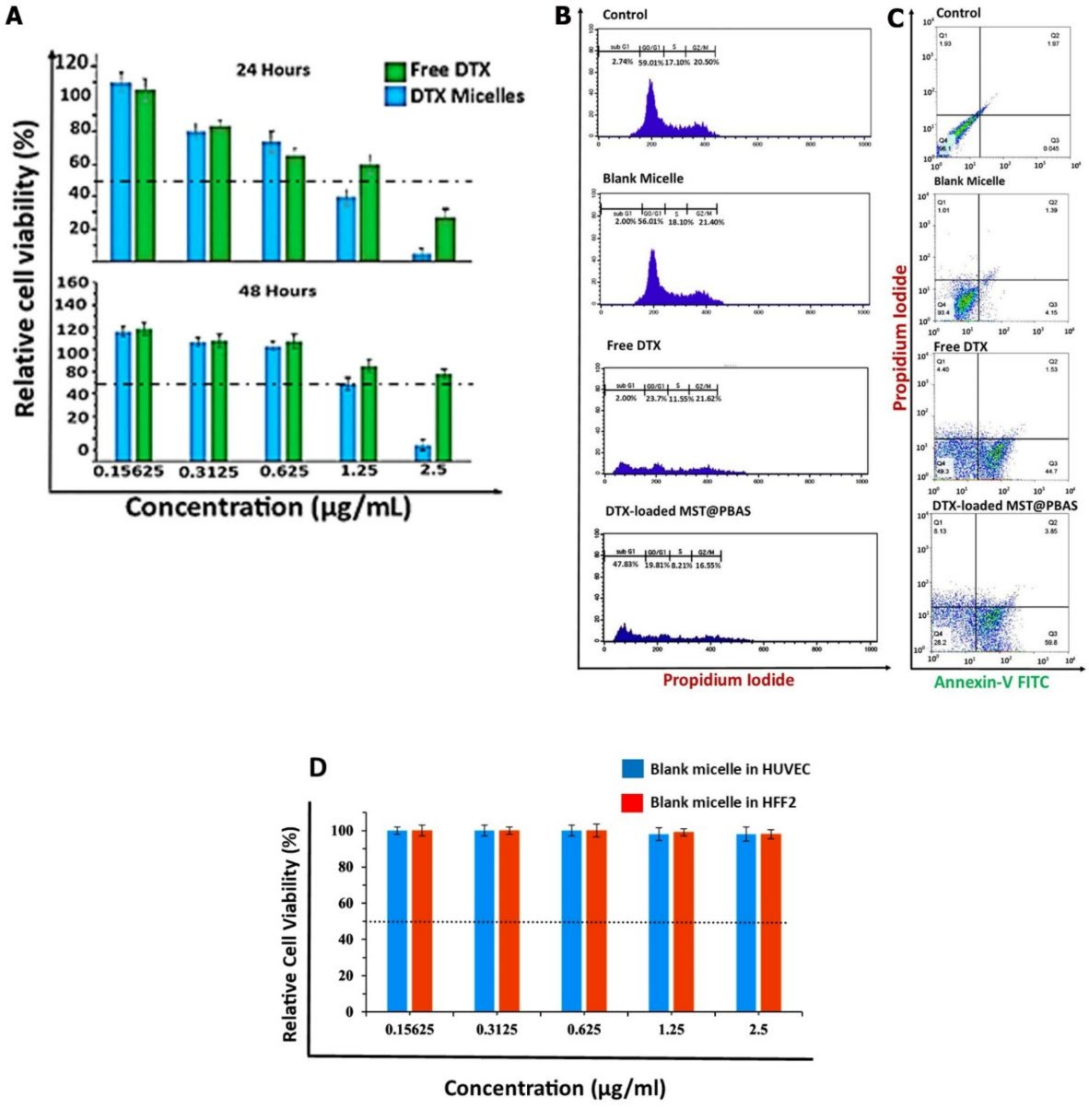
Cell cycle, apoptosis, and in vitro cytotoxicity assay. (**A**) The viability of 4T1 cells was assessed using the MTT assay by treating them with different doses (2.5, 1.25, 0.625, 0.312, 0.156, and 0.078 µg/ml) of free DTX and DTX-loaded MST@PBAS NPs for 24 and 48 h. (**B**) Flow cytometry was used to analyze cell cycle arrest. The effects of blank micelles, free DTX, and DTX-loaded MST@PBAS micelles on the sub G1, G0/G1, S, and G2/M phases were evaluated. (**C**) The apoptosis assay was performed in different formulations and measured by a flow cytometer. The percentages of necrosis, late apoptosis, early apoptosis, and healthy cells are shown in Q1, Q2, Q3, and Q4, respectively. (**D**) The viability of blank micelles in HUVEC and HFFF2 cell lines. mean ± SD, *n* = 3, **p < 0.01; ***p < 0.001.

DTX is a chemotherapeutic agent widely used in the treatment of various cancers, including breast, prostate, and lung cancer. It is a taxane, functioning primarily by stabilizing microtubules and thereby inhibiting cell division, leading to apoptosis^36^. Despite its effectiveness, resistance to DTX is a significant clinical problem. One of the most well-known mechanisms of resistance involves the overexpression of ATP-binding cassette (ABC) transporters such as P-gp (ABCB1). These transporters actively pump docetaxel out of cancer cells, reducing its intracellular concentration and effectiveness (drug efflux pumps)^37^. Therefore, by harnessing smart drug delivery platforms like the one that we used in this article, the drug resistance can be dramatically diminished or eliminated. So, following the cellular internalization experiments, the in vitro cytotoxicity of different concentrations of free DTX and DTX-loaded MST@PBAS NPs was assessed in 4T1 cells at two different time points, as shown in Fig. 3A. The IC50 value of our final designed formulation and free DTX after 24 and 48 h of exposure was determined to be 1.05 µg/ml, 1.6 µg/ml, 1.5 and 7.5, respectively. The nano-formulated DTX after 24 and 48 h exhibited 1.5 and 5-fold stronger cytotoxicity than free DTX. In the case of free DTX, it was observed that drug resistance mechanisms, such as upregulation of multi-drug resistance transporters, are often present in cancer cells^38^. However, the burst release of the drug from our designed NPs saturated the MDR transporters. As a result, the high concentration of the drug inside the cells overcame the cellular defense mechanisms. The synergistic effect of the two ligands (TA1 and SRL-2), which recognized the cancer cells and subsequently increased the absorption of DTX-loaded MST@PBAS NPs, is responsible for the considerable decrease in the IC50 value. Additionally, cell cycle arrest study using flow cytometry was done to evaluate the effect on 4T1 cell proliferation at various cell cycle phases. As shown in Fig. 3B, there was no discernible difference between the control groups and blank micelles in terms of the cell cycle pattern. This outcome supported the safety of PBAS micelles and was in line with the outcomes of the MTT (Fig. 3D) and apoptotic assays (Fig. 3C). Additionally, a significant difference was seen in the sub G1 phase between free DTX and DTX-loaded MST@PBAS micelles, with the nano-formulated form showing a higher percentage than free DTX (48%) that was consistent with the achieved IC50 values and demonstrated the potency of the nanoformulated system that effectively acted with the lowest amount of IC50 in comparison to IC50 of free DTX. Following exposure to MST@PBAS micelles, a considerable rise in the sub G1 population suggested that apoptosis by cell cycle arrest had occurred. This greater incidence of cell cycle arrest reveals the MST@PBAS micelles’ strong targeting potency. A double labeling experiment using PI and Annexin V-FITC was carried out using 4T1 cells to further evaluate the effect of DTX-loaded MST@PBAS micelles on the stimulation of apoptosis (Fig. 3C). The findings showed that DTX-loaded MST@PBAS micelles had higher levels of early (59.8%) and late (3.85) apoptosis than free DTX, which caused early (44.7% of cells) and late (1.53% of cells) apoptosis. These results further revealed the created micelles’ ability to target specific cells and decrease growth while inducing apoptosis. Together, the results of the apoptosis assays and the analysis of the cell cycle indicate that apoptosis is the main mechanism responsible for 4T1 cell death in our study.

### In vitro tumor penetration study

**Fig. 4:**
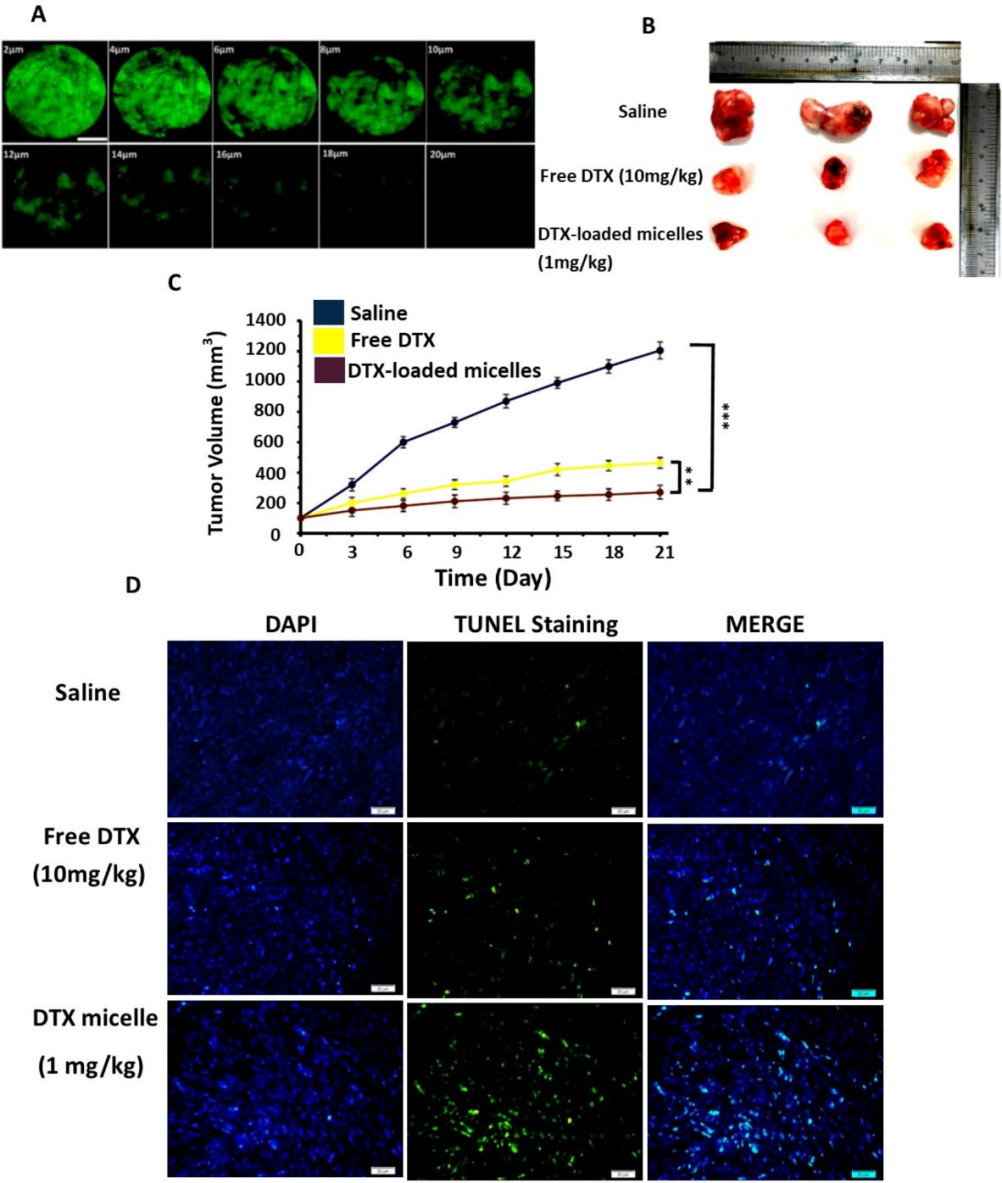
The DTX-loaded micelles’ anticancer properties in vivo. (**A**) Evaluation of the MST@PBAS micelles’ ability to penetrate deep into 3D in vitro tumors. (**B**) Illustration of tumor tissues from 4T1 tumor-bearing mice that were given saline, free DTX, or MST@PBAS NPs that were DTX-loaded. (**C**) A comparison of the tumor responses in tumors treated with saline, free DTX, and MST@PBAS NPs loaded with DTX. (**D**) Assessment of tumor apoptosis using the TUNEL assay after different therapeutic interventions. Mean ± SD, *n* = 3, **p < 0.01; ***p < 0.001.

To assess the penetration depth of MST@PBAS micelles in a 3D in vitro tumor model, FITC-loaded MST@PBAS micelles were utilized as a marker. The 3D tumors were generated and exposed to a concentration of 5 µg/ml of NPs, followed by a 3 hours’ incubation with 4T1 spheroids containing over 2 × 10^4^ cells and measuring 100 μm in diameter. The fixed spheroid was then examined using confocal microscopy, capturing images at 2 μm intervals from the surface to the depth of the tumor. The results revealed a penetration depth of up to 20 μm within the 3D spheroid-formed tumors, which was considered satisfactory compared to the overall tumor size (Fig. 4A). The occurrence of apoptosis within the tumor depth in the subsequent section further supported these findings. The significant penetration depth observed can be attributed to the uptake mechanism of MST@PBAS NPs. As previously mentioned, the sealed paracellular pathway within the TME, characterized by high interstitial pressure and condensed extracellular matrix, renders the transcytosis pathway the most efficient route for reaching the deeper regions of the tumor^39^.

### In vivo antitumor study

Warren C. Chan et al. conducted a meta-analysis study in which revealed that approximately 99% of the administered NPs interacted with off-target sites, while only a small fraction (0.7%) of the NPs could successfully reach the TME. This highlights the challenge of achieving efficient drug delivery to the intended target site. To address this issue, the development of the next generation nanocarriers with a stealth nature, and high accumulation efficiency in the tumor site has become crucial in the field of nanomedicine. By engineering nanocarriers with properties that allow them to evade off-target interactions and efficiently accumulate in the tumor, researchers aim to enhance the specificity and efficacy of drug delivery systems. To evaluate the in vivo antitumor activity, the therapeutic efficacy of DTX-loaded MST@PBAS micelles was compared to free DTX in a 4T1 breast cancer model. Surprisingly, even though superthe dose of DTX administered in the nano-formulated micelles was only 1/10th of the therapeutic dose of free DTX (1 mg/kg), both treatments exhibited similar effectiveness in shrinking tumor size (Fig. 4B). The tumor volume curve made it very evident that during the observation period, the PBS-treated control group had rapid tumor growth. Nevertheless, despite the lower dose, the group receiving treatment with DTX-loaded MST@PBAS micelles shown a greater suppression of tumor growth than the free DTX group. These findings show that the nano-formulated micelles have greater therapeutic activity (Fig. 4C). Additionally, the DTX-loaded MST@PBAS micelles with just 1/10th of TD of free DTX, produced larger necrotic and apoptotic areas, according to analysis of the tumor using the TUNEL assay (Fig. 4D). This can be attributed to the precise penetration, delivery, and targeting capabilities of the designed micelles. The prolonged circulation time of the micelles, facilitated by their stealth feature and biological transformation in the TME triggered by MMP-9, played a significant role. Additionally, the pH/GSH-responsive nature of the engineered micelles enabled enhanced DTX release in the TME, further contributing to their therapeutic efficacy. The presence of two ligands, SRL-2 and TA1, on the surface of the micelles significantly enhanced their targeting ability towards 4T1 cancer cells. The targeting of LRP-1 by the SRL-2 peptide facilitated transcytosis and penetration deep into the tumor. Once at the target site, the unique structure of the micelles underwent destruction in response to the high GSH concentration and low pH of the TME, led to enhanced DTX release. These findings highlight the success of the designed micelles in overcoming issues such as PC formation, and mononuclear phagocyte system entrapment, ultimately resulting in a significant reduction in the TD of DTX. The engineered micelles exhibit higher accumulation capacity and superior therapeutic efficacy, demonstrating their potential as a promising approach in cancer treatment.

### Histological study

**Fig. 5:**
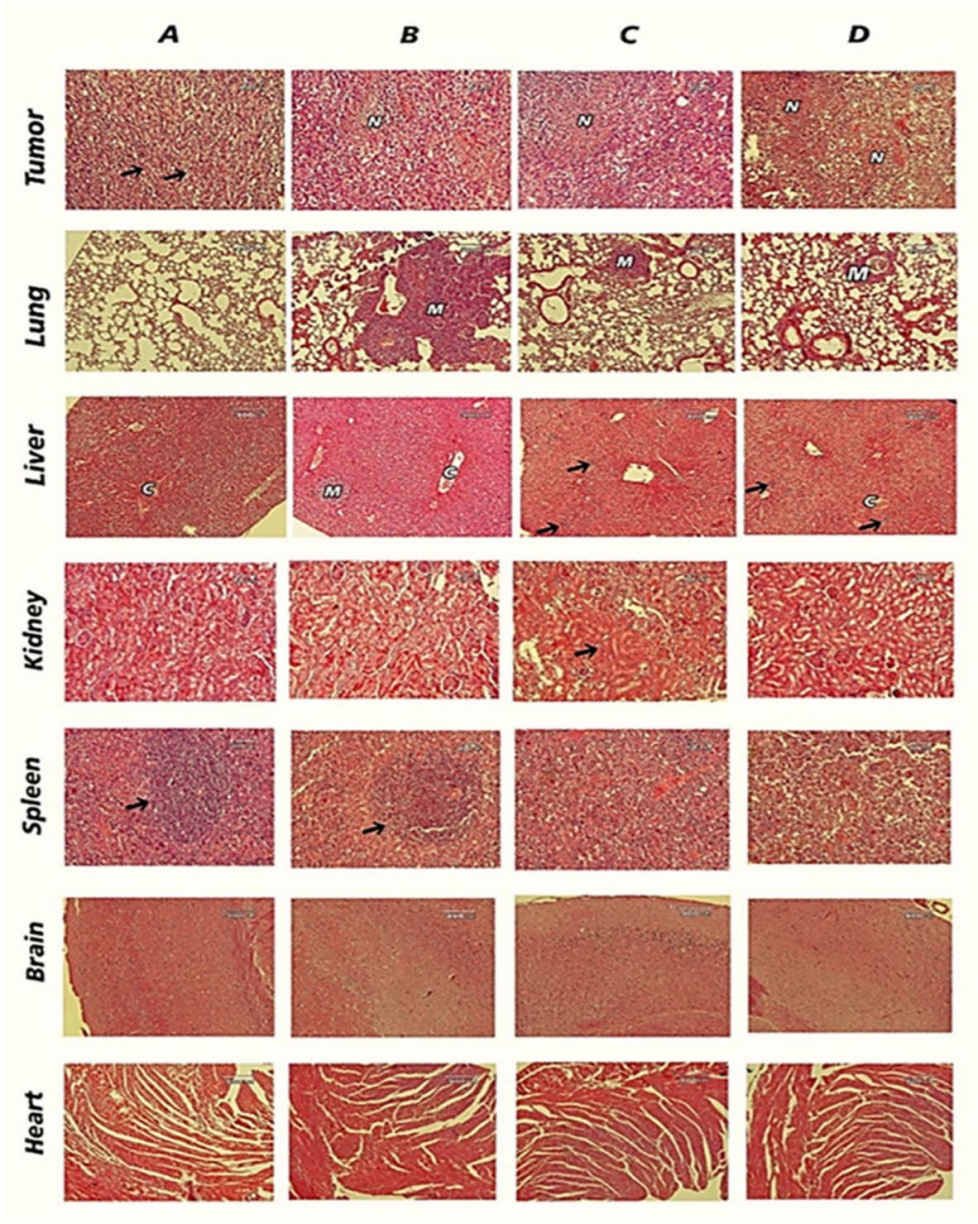
On day 21 following implantation, key organs (heart, kidney, liver, lung, spleen, and brain) and samples of the 4T1 breast cancer tumor were obtained. With the use of a light microscope (Olympus-CH30, Japan), the tissue samples were examined. The histology findings are shown, with each group given the appropriate label: A control-established group, a saline group, a group without DTX, a group with DTX, and a group of DTX-loaded MST@PBAS NPs. The observed features in the tissue sections included metastases (M), necrosis (N), and congestion (C).

Histological evaluation of tumor tissues stained with hematoxylin and eosin (H&E) confirmed the antitumor efficacy. The findings, depicted in Fig. 5, revealed notable differences among the treatment groups. In the saline group, severe malignancy was observed (indicated by arrows), which was significantly attenuated after treatments. The saline and control-established groups’ tumor masses had characteristics of aggressive breast cancer. The severity of the widespread necrosis in the tumor masses in the treated groups (DTX and DTX-loaded MST@PBAS) exceeded that seen in the control groups. Additionally, widespread metastases (shown by arrows) were present in the liver and lung tissue sections of the saline group, accompanied with necrosis, vascular congestion, and bleeding. Microscopic metastases to the lung and liver, along with other pathological lesions, were dramatically decreased in the treated groups, especially the DTX-loaded MST@PBAS group. In comparison to the control groups, similar decreases in germinal centers were seen in the spleen sections of the treatment groups (DTX and DTX-loaded MST@PBAS). Mild acute tubular necrosis (indicated by arrows) was observed in the kidney sections of the DTX group, while no pathological lesions were observed in the heart and brain sections of all groups.

## Conclusion

In this study, we created a brand-new class of physiologically transformable NPs (MST@PBAS) that perform differently when exposed to overexpressed MMP-9 in the TME of breast cancer. The IC50 value of DTX-loaded MST@PBAS micelles and free DTX after 48 h of exposure was determined to be 1.5 µg/ml and 7.5 µg/ml, respectively. The nano-formulated DTX exhibited cytotoxicity that was 5-fold stronger than free DTX (Pvalue˂0.001). Cell cycle assay test results showed that following exposure to MST@PBAS micelles, a considerable rise in the sub G1 population (48%) suggested that apoptosis by cell cycle arrest had occurred. DTX-loaded MST@PBAS micelles revealed significantly higher (Pvalue˂0.001) levels of early apoptosis (59.8%) than free DTX (44.7%). Surprisingly, the DTX-loaded MST@PBAS micelles with just 1/10th of TD of free DTX, produced larger necrotic and apoptotic areas, according to analysis of the tumor using the TUNEL assay. Histological study results showed that, the severity of the widespread necrosis in the tumor masses in the DTX-loaded MST@PBAS treated groups exceeded that seen in the free DTX and control groups. Microscopic metastases to the lung and liver, along with other pathological lesions, were dramatically decreased in the DTX-loaded MST@PBAS treated groups compared to free DTX and control groups. This success can be due to the modified NPs’ camouflage properties, which allowed them to avoid RES absorption and reduce off-target accumulation, as shown by in vivo imaging. Upon accumulation in tumor tissues, the redox-responsive linker within the NPs underwent cleavage in response to the high redox potential of the 4T1 cells. This triggers the disassembly of the nanocarrier structure, leading to the rapid release of the encapsulated DTX due to the low pH and high GSH concentration within the 4T1 cells Our findings highlight the dual targeting nature of DTX-loaded MST@PBAS NPs, as well as the synergistic effect of the two ligands incorporated in their design, along with the pH/GSH-mediated drug release. Based on our results, DTX-loaded MST@PBAS NPs hold great promise as a superior alternative for cancer treatment compared to other existing platforms. The ability to enhance circulation time, improve targeting efficiency, facilitate precise drug delivery, and enhance therapeutic efficacy makes these NPs highly desirable for future clinical applications in cancer chemotherapy.

## Data Availability

No datasets were generated or analysed during the current study.
